# Patient-Specific Dual-Function 3D-Printed Gels for Antimicrobial and Analgesic Management of Alveolar Osteitis

**DOI:** 10.1021/acsmaterialsau.5c00226

**Published:** 2026-04-06

**Authors:** Mateo Dallos Ortega, Vahid Heravi Shargh, Jenny Aveyard, Mark Hunter, Alexander Ciupa, Raechelle A. D’Sa

**Affiliations:** † School of Engineering, 4591 University of Liverpool, Harrison Hughes Building, Brownlow Hill, Liverpool L69 3GH, U.K.; ‡ Materials Innovation Factory, 4591 University of Liverpool, 51 Oxford Street, Liverpool L7 3NY, U.K.

**Keywords:** 3D-printing, gelatin gels, biomaterial inks, antimicrobial, alveolar osteitis

## Abstract

Alveolar osteitis (AO), or dry socket, is a painful postoperative complication caused by the premature loss of the blood clot from the extraction site. Current treatments rely on irrigation and temporary dressings, however these approaches offer limited therapeutic benefits and often require repeated interventions. This work describes a customizable, dual-function biomaterials platform based on 3D-printed gelatin gels incorporating the antimicrobial quaternary ammonium compound, benzyldimethyldodecylammonium (BDMDAC), and the analgesic lidocaine enabling sustained infection control and localized pain management. 3D-printed gels were optimized for printability, mechanical stability, and controlled drug release, and their physicochemical properties were characterized through swelling, degradation, and release studies. Both hydrated and freeze-dried gels were evaluated to assess how architecture influences transport behavior and therapeutic performance. Antimicrobial efficacy was evaluated against clinically relevant oral pathogens, including *Porphyromonas gingivalis*, *Enterococcus faecalis*, and *Streptococcus mutans* which are key pathogens associated with oral infections. Cytocompatibility and inflammatory responses were assessed using human gingival fibroblasts (HGF-1s) as well as Interleukin-6 (IL-6) and tumor necrosis factor (TNF-α) expression. The 3D-printed gels sustained antimicrobial activity, achieving complete planktonic pathogen eradication and biofilm inhibition within 24 h, independent of lidocaine incorporation. Analysis of pro-inflammatory cytokine markers showed a minimal response in most gel formulations, with a slight increase at high lidocaine concentrations (30 mg/mL), whereas freeze-dried gels produced a more pronounced early inflammatory response at this concentration. Finally, 3D printed anatomical patient-specific molar-shaped gels preserved antimicrobial efficacy comapred with grid-printed controls, confirming that therapeutic performance is maintained across complex geometries. Overall, these results demonstrate that on-demand fabrication of patient specific, antimicrobial, and analgesic 3D printed gels has the potential to be used for localized management of AO.

## Graphical abstract

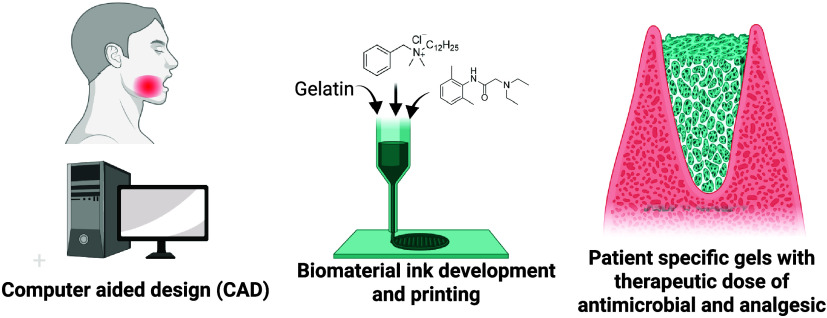

## 1 Introduction

Alveolar osteitis (AO), or “dry socket”, is a painful complication that typically arises within 1 to 3 days following a difficult or traumatic tooth extraction. It occurs when the protective blood clot within the extraction socket becomes dislodged or dissolves prematurely, leaving the underlying alveolar bone exposed.^1^ This exposure leads to persistent pain and inflammation, delaying healing and causes significant patient discomfort.^1,2^ Tooth extraction is a routinely performed dental procedure worldwide. A large scale study in Switzerland of 17,784 of patients aged 15 to 74 years reported that extraction represented 5.4% of all dental treatments.^3^ Similarly, an epidemiological survey in Brazil found that permanent tooth extractions accounted for 10.2% of all dental procedures performed between 1998 and 2012 (*n* = 161,812,852).^4^ Although, AO affects only 1–5% of routine extractions, its incidence increases dramatically to 30% for wisdom teeth, underscoring its clinical relevance.^5^

Although the precise pathogenesis of AO remains unclear, the Birn’s theory of localized fibrinolysis is widely cited, proposing that increased fibrinolytic activity at the extraction site results in premature blood clot dissolution.^2,6^ In addition, multiple risk factors have been implicated in the development of AO, including traumatic or forceful extraction, pre-existing infection or inflammation of the socket, excessive vasoconstrictor use in local anesthetics, mechanical dislodgement of the clot due to vigorous rinsing, hormonal influences such as oral contraceptive use, and heavy smoking.^7,8^

Current treatment strategies focus on symptomatic relief, aiming to alleviate symptoms rather than address the underlying pathology. Standard management includes gentle irrigation to remove debris and necrotic tissue followed by the placement of intrasocket medicaments comprising antibiotics, topical anesthetics, and analgesics. Commonly used agents include zinc oxide and eugenol, Alvogyl (eugenol, iodoform, and butamben), metronidazole and lidocaine ointment. More advanced interventions such as low-level laser therapy and platelet-rich fibrin are also being explored to accelerate healing and improve patient outcomes.^2,9,10^

A variety of biomaterials, including allografts, xenografts, and synthetic products (alloplasts), have been commonly used in alveolar socket repair. However, these approaches are associated with several challenges, such as limited graft availability, suboptimal cytocompatibility, inadequate osteointegration, and the need for multiple dental visits for irrigation and dressing changes.^2,11^ In contrast, gels represent a promising next-generation strategy due to their excellent biocompatibility, high drug-loading and release capacity, structural similarity to the extracellular matrix, and tunable mechanical properties.^12,13^ In addition to regenerative applications, gel systems have been explored as localized antimicrobial delivery platforms. For example, gelatin emulsion gels loaded with host defense peptides have demonstrated effective eradication of antibiotic-resistant bacteria and biofilms while maintaining cytocompatibility, highlighting the potential of gel-based matrices for treating infected soft tissues.^14^ Zhang et al. developed a multifunctional bone repair strategy in which magnesium ascorbyl phosphate was incorporated into gelatin methacrylate (GelMA) hydrogels for sustained release for regenerative applications. The scaffold promoted bone regeneration by supplying phosphorus-containing compounds without the need for exogenous calcium. MAP enhanced bone repair by reducing oxidative stress in bone marrow-derived mesenchymal stem cells, promoting calcium uptake, and accelerating mineralization.^15^

Recently, our group developed a 3D printed gelatin gel scaffold incorporating the quaternary ammonium compound, benzyldimethyldodecylammonium (BDMDAC) for application in regenerative endodontics.^16^ The scaffold exhibited sustained release of the BDMDAC over 60 h and demonstrated excellent antimicrobial efficacy against three clinically relevant oral pathogens including *Porphyromonas gingivalis*, *Enterococcus faecalis* and **Streptococcus mutans*.* While this approach could also be promising for AO, the real breakthrough lies in patient-specific drug delivery gels engineered to deliver antimicrobial and analgesic agents precisely at the extraction site. Unlike conventional “one-size-fits-all” formulations, 3D printing offers the possibility of truly personalized drug delivery. The integration of anatomical precision with programmable, localized drug release, enables 3D-printed gels to bridge critical gaps between scaffold design, pharmacotherapy, and individualized patient care. It represents a natural evolution in personalized medicine, shifting treatment from generalized symptom relief to targeted, precision-based therapy. This convergence is particularly advantageous in conditions like AO, where both defect-specific scaffold geometry and controlled dual drug delivery are essential for effective clinical outcomes.

In this paper, we hypothesized that patient-specific dual-function 3D printed gels could be engineered to provide localized, controlled release of therapeutic agents capable of simultaneously preventing bacterial infection and alleviating postoperative pain. To test this hypothesis, BDMDAC and lidocaine were incorporated into 3D-printed gelatin gels designed for the treatment of AO. The biomaterial inks were optimized to ensure suitable printability, shape retention, and mechanical stability. The physicochemical and mechanical properties were evaluated in terms of swelling, degradation, and drug release profiles, with HPLC used to determine release kinetics. The antimicrobial efficacy of the gels, including both planktonic cell inhibition and suppression of biofilm development, was tested against *P. gingivalis*, *E. faecalis* and *S. mutans.* Their cytocompatibility was assessed using human gingival fibroblasts (HGF-1s). Additionally, to investigate potential inflammatory reactions triggered by the gels, the levels of interleukin-6 (IL-6) and tumor necrosis factor-alpha (TNF-α) were measured by an enzyme-linked immunosorbent assay (ELISA) (Figure 1). Finally, the proof-of-concept for patient-specific gels was demonstrated by fabricating anatomical molar-shaped gels for patients of different sizes (adult and pediatric). Drug loading was optimized to maintain antimicrobial and analgesic efficacy across varying sizes, and these gels were tested for their feasibility of delivering consistent therapeutic outcomes independent of patient anatomy whist maintaining their physicochemical properties.

**Figure 1:**
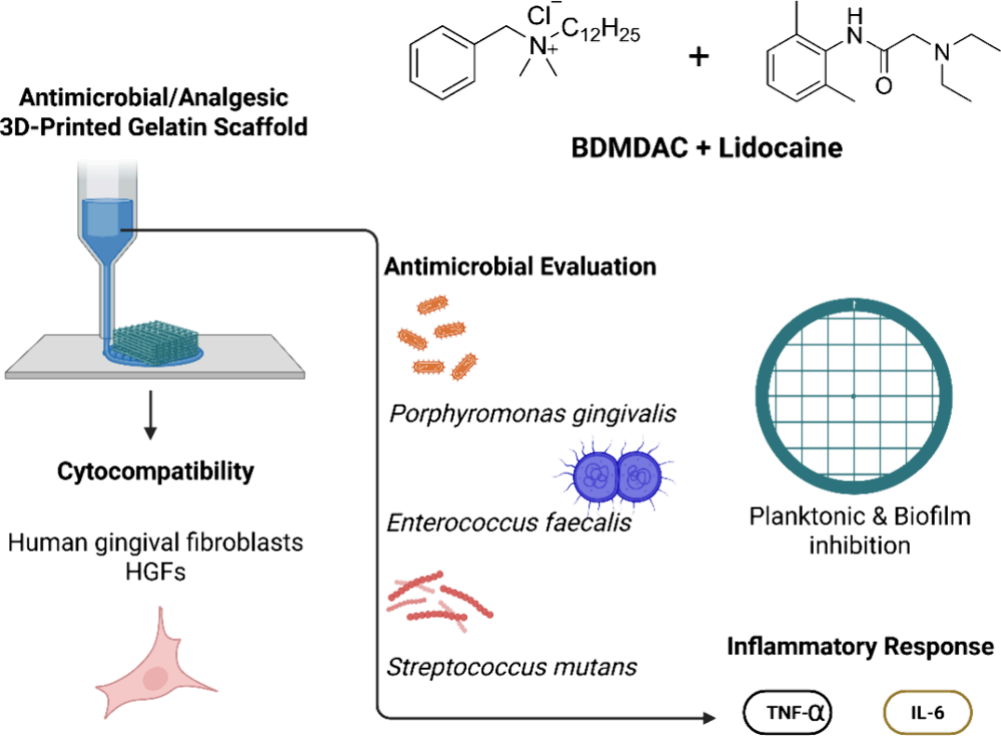
Schematic overview of the experimental workflow of dual action 3D printable biomaterial ink for AO treatment.

## 2 Materials and Methods

### 2.1 Materials

Gelatin A from bovine skin (150 bloom), benzyldimethyldodecylammonium chloride (BDMDAC), glutaraldehyde (50 wt % in H_2_O solution), ethanol, nutrient agar (NA), nutrient broth (NB), Luria Broth (LB), Luria Agar (LA), tryptic soy broth (TSB), tryptic soy agar (TSA), menadione (vitamin K3), hemin from porcine, and lidocaine hydrochloride were purchased from Sigma-Aldrich. Columbia broth (CB), Columbia agar (CA) and defibrinated sheep blood were sourced from Darwin Biological.

### 2.2 Gelatin-BDMDAC-Lidocaine Biomaterial Ink Preparation

A 5% w/w gelatin biomaterial ink (GL5) was prepared by dissolving 0.25 g of gelatin in 5 g of distilled water at 60 °C and stirring at 600 rpm for 1 h until a homogeneous solution was obtained. For gelatin inks loaded with BDMDAC and lidocaine (GL5-Q125-L), two lidocaine concentrations (15 and 30 mg/mL) and a single concentration of BDMDAC (125 μg/mL) were added to preheated water at 60 °C and stirred at 600 rpm for 1 h. The resulting samples were designated as follows: GL5-Q125-L15 (125 μg/mL BDMDAC and 15 mg/mL lidocaine) and GL5-Q125-L30 (125 μg/mL BDMDAC and 30 mg/mL lidocaine). Before printing, the inks were loaded into 4 mL cartridges and kept at room temperature for 30 min. The nomenclature and conditions for all sample types are summarized in Table 1. GL5 was used solely for rheology characterization as a baseline comparison. Its antimicrobial efficacy and cell viability have been previously assessed.^17^

**Table 1:** Nomenclature of Gel (GL-) and Freeze Dried (FGL-) Gelatin-BDMDAC/Lidocaine Biomaterial Ink Composition

**sample name**	**BDMDAC**concentration (μg/mL)	**lidocaine** **concentration**(mg/mL)
GL5		
GL5-Q125	125	
GL5-Q125-L15	125	15
GL5-Q125-L30	125	30
FGL5-Q125	125	
FGL5-Q125-L15	125	15
FGL5-Q125-L30	125	30

### 2.3 3D Printing

The 3D-printed gels were designed using 3D CAD software (Creo Parametric) to create stereolithography (STL) files, which were then converted into G-code using Slic3r, an integrated tool within Cellink’s operating software (DNA). During the slicing process, key printing parameters such as layer height, printing speed, and infill percentage were defined. Among these, the infill percentage is particularly crucial, as it determines pore size by controlling the spacing between adjacent filaments. Gels were fabricated using a dual-printhead extrusion-based 3D printer (BIOX6, Cellink) equipped with a 27G nozzle. Printing was conducted at 28 °C with a pressure range of 100–115 kPa and a speed of 10 mm/s. The final construct consisted of 12 layers, each 0.2 mm thick, with an infill percentage of 20%, resulting in a 2.5 mm filament spacing. The printed gels measured 10 × 10 × 3 mm. Postprinting, the gels were cross-linked in a 0.5% glutaraldehyde solution for 30 min while being agitated at 50 rpm. They were then washed three times with distilled water, each wash lasting 5 min. To ensure sterility, the gels were exposed to UV light (265 nm) for 30 min before use. To enhance durability and longevity, freeze-drying (lyophilization) was performed for 24 h using a freeze-dryer (Scanvac CoolSafe Touch 110–4, Denmark). Freeze-dried gels were included to reflect a clinically relevant storage format with extended shelf life, as previously validated by micro computed tomography analysis demonstrating preserved porosity and architecture after rehydration.^16^ Comprehensive rheological and printability characterization of the gelatin based biomaterial inks is provided in the Supporting Information. These analyses validate the suitability of the formulations for extrusion-based 3D printing and support the printing parameters used in this study. Oscillatory rheology was employed to assess viscoelastic behavior, thermal stability, and shear thinning properties, confirming that all formulations exhibit predominantly solid like behavior with enhanced structural stability upon incorporation of BDMDAC and lidocaine (Supplementary Figure S1). In addition, filament collapse, diffusion rate, and printability analyses were performed to quantitatively evaluate shape fidelity and gelresolution across different grid spacings (Supplementary Figures S2 and S3). The supplementary data demonstrate that the incorporation of antimicrobial and analgesic agents does not compromise printability or structural integrity, thereby supporting the feasibility of patient specific gel fabrication reported in the main text.

### 2.4 Swelling and Degradation

The swelling behavior of the gels was assessed by immersing the samples in sterile PBS at 37 °C for 72 h, with measurements recorded every 6 h. The swelling rate (*Sw*%) was determined using the following formula:^18,19^

$$Sw\%=\frac{W_{w}-W_{d}}{W_{d}}\times 100$$ 1

where *W*_w_ and *W*_d_ represent the weights of the gels in its swollen and dry states, respectively. Excess water was gently removed using laboratory paper towels before measurement. All experiments were conducted in triplicate.

The degradation rate was assessed by immersing the samples in PBS and incubating them at 37 °C. At 6-h intervals, up to a total of 72 h, the gels were removed, dried, and weighed. The remaining gel weight after degradation was calculated using the following equation:^18,19^

$$\mathrm{Weight}\;\mathrm{remaining}(\%)=100-\left[\frac{W_{0}-W_{f}}{W_{0}}\times 100\right]$$ 2

where *W*_0_ is the initial weight of the gel and *W*_f_ is the final weight of the gel.

### 2.5 BDMDAC-Lidocaine Release

The release profiles of BDMDAC and lidocaine from the gelatin-BDMDAC-lidocaine gels were analyzed using high-performance liquid chromatography (HPLC) with an Agilent Technologies 1260 Infinity II System (Radnor, USA) under isocratic conditions at room temperature. A 4.6 × 250 mm ZORBAX SB-C18 analytical column was utilized for BDMDAC detection, while a 2.1 × 150 mm ZORBAX SB-CN column was used for lidocaine detection.

Gels were immersed in 1 mL of deionized (DI) water at 37 °C for varying time intervals. After incubation, each aliquot was centrifuged at 4000 × *g* for 15 min, and any undissolved gelatin-BDMDAC-lidocaine was filtered using a protein concentrator. Both analyses used an 80% acetonitrile (in 0.1% formic acid) and 20% water mobile phase, with a flow rate of 1 mL/min. Detection was performed at 254 nm with an 8 nm bandwidth. A G7129A autosampler injected 50 μL of each sample, and concentrations were quantified based on the peak area measurements.

### 2.6 Antimicrobial Assay (Planktonic)

Overnight cultures of *P. gingivalis* NCTC 11834, *S. mutans* NCTC 10449, and *E. faecalis* NCTC 13779 were prepared by incubating *P. gingivalis* and *E. faecalis* in TSB supplemented with hemin (5 μg/mL) and menadione (1 μg/mL) for 60 and 24 h, respectively. *S. mutans* was cultured in CB at 37 °C for 24 h. The bacterial suspensions were adjusted to a final concentration of 10^6^ colony-forming units per milliliter (CFU/mL) using a 0.5 McFarland Standard.^20^ 3D-printed gels were inoculated with 1 mL of the diluted cultures and incubated in a shaking incubator at 37 °C for 4 and 24 h. *P. gingivalis* samples were maintained under anaerobic conditions. At each time point, the incubated samples were serially diluted and plated on TSB blood agar for *P. gingivalis* and *E. faecalis* and Columbia Blood agar for *S. mutans* using the Miles and Misra method to determine CFU counts.^21^ The antimicrobial efficacy of the 3D printed gels was assessed by measuring the reduction in CFUs over the 4 and 24 h time periods.

### 2.7 Biofilm Inhibition Assay

For the biofilm inhibition assay, samples were inoculated with diluted bacterial cultures following the same procedure as the planktonic assays. The inoculated samples were incubated in a shaking incubator at 37 °C for 24 h, with *P. gingivalis* was maintained under anaerobic conditions. After incubation, nonadherent bacteria were removed by gently rinsing the samples with PBS. Biofilms were then detached and resuspended by sonicating the samples for 15 min in 1 mL of TSB or CB. Bacterial CFUs were quantified by serially diluting the suspensions and plating them using the Miles and Misra method on TSB blood agar for *P. gingivalis* and *E. faecalis* , and Columbia Blood agar for*S. mutans*

### 2.8 Cell Viability

Human gingival fibroblast cells (HGF-1) (ATCC CRL-2014) were cultured in ATCC-formulated Dulbecco’s Modified Eagle’s Medium (Catalog No. 30–2002), supplemented with 10% fetal bovine serum (Thermo Fisher Scientific, USA), at 37 °C and 5% CO_2_. Extracts from the 3D printed gels and controls were collected at 24 post incubation in the supplemented cell culture medium, following ISO 10993 guidelines for testing medical devices.^22^ HGF-1 cells were seeded in 96-well plates (Corning Costar, Flintshire, UK) at a density of 1 × 10^4^ cells/well and then replaced with 100 μL of the extracted medium from the printed gels and controls, followed by an additional 24 and 72 h incubation. A stock MTT (Thiazolyl Blue Tetrazolium Bromide, Sigma) solution of 5 mg/mL was prepared in PBS and sterile-filtered. The medium in each well was aspirated, and 100 μL of the MTT working solution (1 mg/mL) in culture medium was added to each well, followed by incubation at 37 °C. After 4 h, 100 μL of dimethyl sulfoxide (DMSO) was added to each well, and the plate was wrapped in aluminum foil and shaken on an orbital shaker for 10 min. The absorbance signal was measured at 570 nm using a Synergy HT Plate Reader (BioTek, Agilent Technologies, Inc., USA). The background absorbance was subtracted from the signal absorbance (OD_570_ - OD630) to obtain normalized absorbance values. Percentage cell viability was calculated as the ratio of the normalized values for each treatment to that of control cells, multiplied by 100. The experiment was repeated three times with four technical replicates.

### 2.9 Quantitation of the TNF-α and IL-6 Release from HGF-1 Cells by Means of Enzyme-Linked Immunosorbent Assay (ELISA)

The release of IL-6 and TNF-α from cell cultures was assessed using an ELISA assay, following the manufacturer’s protocol (Invitrogen EH2IL6 and 88–7346). Aliquots of supernatant (100 μL) were collected from the exposed media at 1 and 6 h, and the pro-inflammatory cytokine levels were quantified by comparing the measured values to standard curves constructed with purified human IL-6 and TNF-α.

### 2.10 Molar-Shaped Gel Fabrication and Mass Normalization

Grid-pattern gels were fabricated using extrusion-based 3D printing from a 4 mL cartridge containing 625 μg of BDMDAC and 75 mg of lidocaine dissolved in 5 g of water. Six gels were produced per cartridge, corresponding to approximately 0.667 g of extruded material per construct. Drug loading was therefore calculated based on the total mass of extruded material, resulting in theoretical values of 104 μg BDM and 12.5 mg lidocaine per gel.

To ensure comparability across gel geometries, anatomical molar-shaped gels were fabricated with controlled infill percentages to match the mass of the grid-pattern gels. Adult molar gels were printed with an infill of 25% using identical footprint and height parameters as the grid gels. Pediatric molar gels were printed with proportionally reduced footprint and height to mimic pediatric socket dimensions, and the infill was adjusted to 30% to achieve comparable mass. The wet mass of three representative gels was measured for each gel type immediately after printing to confirm reproducibility and consistency with theoretical material usage. Measurements were performed using an analytical balance with a precision of ±0.1 mg. The dimensions of the adult molar gels were designed to approximate the average root geometry of a human permanent molar, excluding the crown portion. Anatomical studies report mesiodistal root spans of 10–12 mm, buccolingual spans of 9–11 mm, and root lengths of 13–15 mm.^23^ The final adult gel footprint (11.86 × 10.54 mm) and height (14.26 mm) closely match these parameters, providing a clinically relevant representation of the extraction socket. For pediatric applications, the gel was uniformly scaled to 94% of the adult size, resulting in a footprint of 11.15 × 9.91 mm and height of 13.40 mm, which falls within the range of primary molar root dimensions. The infill was adjusted (25% for adult, 30% for pediatric) to normalize mass and drug loading across gel types, ensuring comparable therapeutic performance independent of geometry (Table 2).

**Table 2:** Gel Grid Dimensions, Values Represent Wet Mass Measured Immediately after Fabrication (*n* = 3)

**geltype**	**infill (%)**	**measured masses (mg)**	**mean ± SD (mg)**	**footprint (mm) (*X* × *Y*)**	**height (mm)**	**notes**
grid-pattern	20	685, 703, 705	698 ± 11	10 × 10	3	baseline gel used for optimization and antimicrobial testing
adult molar	25	679, 677, 670	675 ± 5	11.86 × 10.54	14.26	anatomic gel for mandibular molar socket
pediatric molar	30	650, 660, 647	652 ± 7	11.15 × 9.91	13.4	reduced-size gel pediatric molar socket

The antimicrobial planktonic and biofilm assays were performed on these gels following methods Sections 2.6 and 2.7, respectively.

### 2.11 Statistical Analysis

One-way analysis of variance (ANOVA) was used to compare whether the difference in antimicrobial efficiency and other measurements between each bioprinted sample was significantly different. A value of *p* < 0.05 was taken as being statistically significant.

## 3 Results and Discussion

In this work we have fabricated 3D printed dual-function gels for antimicrobial and pain management of AO. The biomaterial inks for 3D printing were optimized in terms of rheology for optimal physicochemical and mechanical properties. The biological response was tested in term of antimicrobial efficacy, cytotoxicity and anti-inflammatory response. Comprehensive rheological and printability characterization of the gelatin based biomaterial inks is provided in the Supporting Information. These analyses validate the suitability of the formulations for extrusion-based 3D printing and support the printing parameters used in this study. Oscillatory rheology was employed to assess viscoelastic behavior, thermal stability, and shear thinning properties, confirming that all formulations exhibit predominantly solid like behavior with enhanced structural stability upon incorporation of BDMDAC and lidocaine (Supplementary Figure S1). In addition, filament collapse, diffusion rate, and printability analyses were performed to quantitatively evaluate shape fidelity and gel resolution across different grid spacings (Supplementary Figures S2 and S3). Overall, this data demonstrates that the incorporation of antimicrobial and analgesic agents did not compromise printability or structural integrity, thereby supporting the feasibility of patient specific gel fabrication reported in the main text.

### 3.1 Swelling and Degradation

The swelling and degradation behavior of the 3D printed gelatin gels were consistent across all formulations, indicating that incorporation of BDMDAC and licocaine had minimal impact on structural integrity. Swelling studies (Figure 2a,b) demonstrated that all gels absorbed a significant amount of water over time due to their hydrophilic nature.^24–26^ The gelatin gels (GL5, GL5-Q125, GL5-Q125-L15, and GL5-Q125-L30) reached approximately 100% swelling after 25 h, whereas freeze-dried gels (FGL5, FGL5-Q125, FGL5-Q125-L15, and FGL5-Q125-L30) exhibited substantially higher swelling ratios of 2000–2500%, likely due to increased porosity and surface area.^27^ After reaching a maximum, swelling decreased, suggesting structural relaxation or partial dissolution. Weight remaining values exceeding 100% reflect gel swelling due to fluid absorption during immersion, as initial mass was normalized to the pre immersion weight. Degradation studies (Figure 2c,d) further support the notion that the structural stability of the gels remains largely unaffected by the incorporation of BDMDAC and lidocaine.^2^

**Figure 2:**
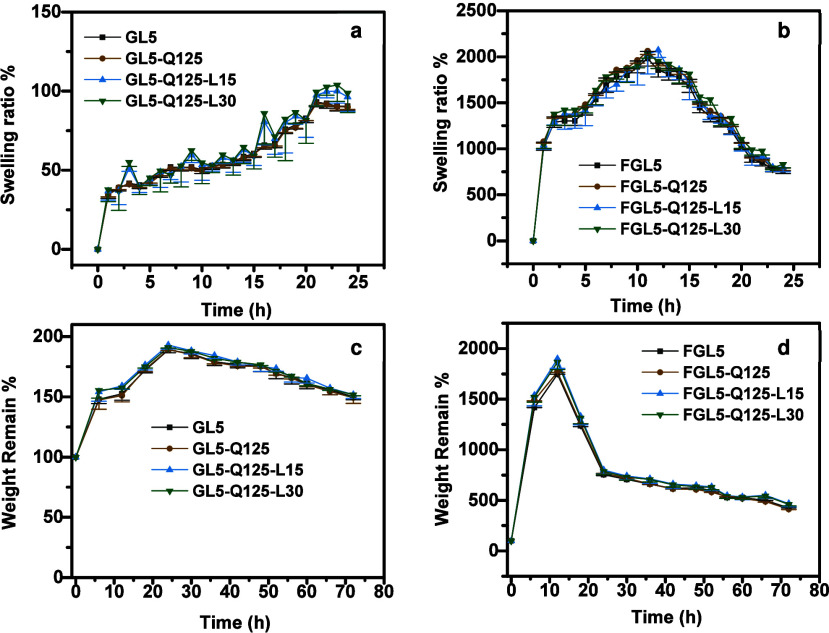
Swelling and degradation behavior of gelatin-based gels with different compositions. (a) Swelling ratio of GL gels over time. (b) Swelling ratio of FGL gels, showing higher water absorption. (c) Weight remaining of GL gels, indicating gradual degradation. (d) Weight remaining of FGL gels follows a similar trend. Data represent the mean ± standard deviation for *N* = 3 independent measurements.

Initially, all GL gels exhibited a sharp increase in weight retention, likely due to rapid water absorption and swelling. This was followed by a peak, after which the weight gradually decreased over time, indicating progressive degradation. The FGL gels exhibited a similar trend, with an initial weight gain followed by a gradual mass loss, suggesting that although they absorb a larger amount of water, their degradation profile remains comparable to that of GL gels. Similar weight loss profiles within each group confirm that BDMDAC and lidocaine do not significantly affect degradation, supporting the use of these gels as stable matrices for controlled drug release. This is a salient point, as it demonstrates that the incorporation of active agents does not compromise the structural integrity of the material over time.

Importantly, these swelling and degradation trends directly inform the subsequent drug release behavior. The rapid swelling and earlier structural relaxation observed for the GL are expected to shorten diffusion path lengths and promote faster initial drug transport, consistent with the earlier release and plateau observed in Section 3.2. In contrast, FGL gels, despite exhibiting substantially higher water uptake due to their porous architecture, retained structural integrity over longer periods, which supports sustained diffusional pathways and prolonged release at later time points. Thus, the temporal differences in drug release kinetics correlate with the distinct degradation and structural evolution profiles of the two gel formats, indicating a coupled diffusion–degradation controlled mechanism.

### 3.2 BDMDAC-Lidocaine Release

The cumulative release profiles of BDMDAC (Figure 3a) demonstrated a gradual and sustained increase in concentration over time for all gels. Consistent with diffusion-controlled transport expected for hydrated gelatin matrices, both GL and FGL showed progressive drug release without an initial burst, indicating that BDMDAC remained well retained within the gels and diffused steadily through the matrix. Most GL gels began to stabilize after approximately 45 h, reaching a plateau by 55 h, while the FGL gels exhibited a similar trend with a clearly defined plateau. This behavior suggests a prolonged diffusion process governed by matrix-controlled transport rather than rapid surface desorption. Such sustained release is likely enabled by the gel network’s capacity to retain water and regulate molecular diffusion, as previously reported for comparable gelatin-based systems.^28^

**Figure 3:**
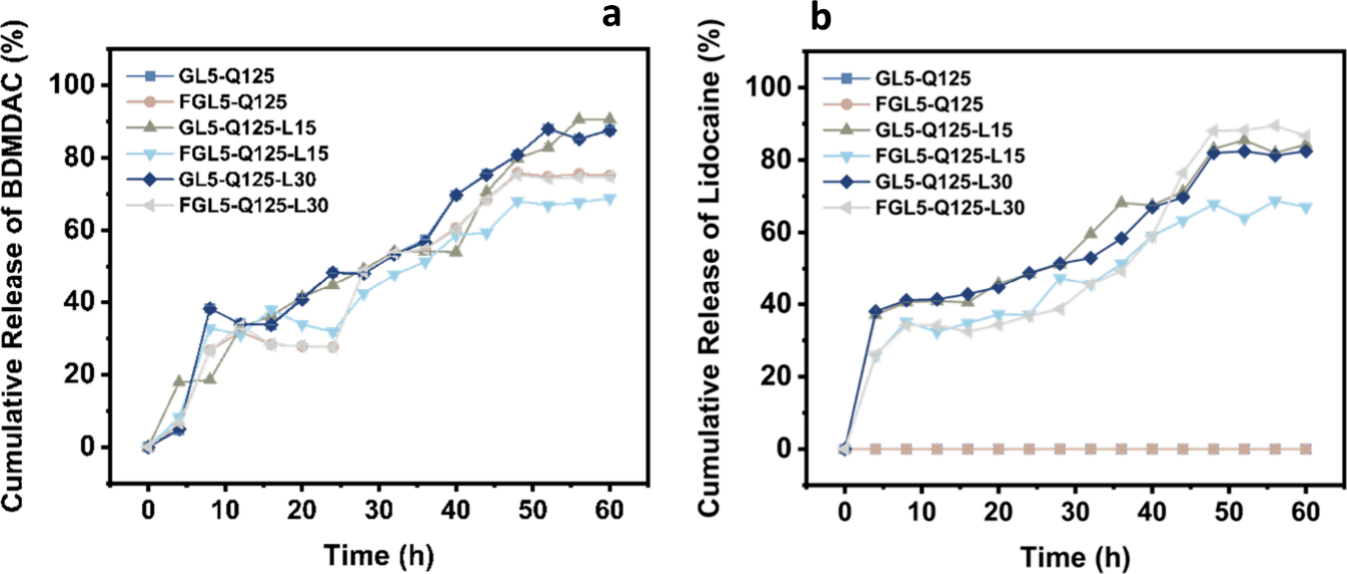
HPLC cumulative release profiles of (a) BDMDAC and (b) lidocaine cumulative release from GL and FGL over 60 h.

In addition to diffusion through the hydrated network, the release behavior is also expected to depend on the degradation and structural evolution of the gels during incubation. Upon rehydration, GL gels likely swells rapidly, forming continuous aqueous pathways that enhance early diffusional transport. However, this rapid swelling may simultaneously accelerate network softening and erosion, progressively reducing structural integrity and shortening effective diffusion path lengths, thereby contributing to earlier stabilization or plateauing of release. In contrast, FGL gels possess a macroporous architecture that rehydrates more gradually and initially restricts molecular transport, yet retains mechanical integrity for longer periods. As degradation proceeds at later time points, pore wall erosion and increased interconnectivity enhance effective diffusivity and sustain drug release. Consequently, drug release is more accurately described by a combined diffusion-degradation mechanism rather than purely Fickian diffusion. This follows from our previous work where we demonstrated freeze-drying generates interconnected porosity that supports sustained transport, while non freeze-dried gels exhibit more limited internal pathways.^16^

The gels containing higher lidocaine concentrations (GL5-Q125-L30 and FGL5-Q125-L30) exhibited greater cumulative release over time, highlighting the influence of initial drug concentration on diffusion (Figure 3b). Based on their increased porosity and surface area, FGL gels would typically be expected to promote faster drug release. However, GL gels showed slightly higher lidocaine release than their FGL counterparts throughout most of the experimental period. This observation suggests that the hydrated gel matrix may facilitate more efficient diffusion, likely due to improved water retention and the presence of a continuous aqueous phase that supports sustained drug transport. Similar behavior has been reported by Simoni et al., who demonstrated that drying methods significantly influence gelatin network structure and diffusion characteristics, with denser, non freeze-dried hydrogels exhibiting distinct swelling and transport properties.^29^

At later time points, however, this trend shifted; after approximately 45 h, the FGL5-Q125-L30 surpassed its GL counterpart in cumulative release. This late-stage increase may be attributed to progressive gel degradation, which reduces structural integrity and limits sustained diffusion, thereby allowing the more porous FGL gel to dominate release. Overall, GL gels favor greater early to-midterm release, whereas FGL gels may provide delayed but sustained drug delivery.

To elucidate the mechanism of drug transport, the cumulative release profiles of BDMDAC and lidocaine were fitted to 0th-order, 1st-order, and Higuchi kinetic models (Table 3). Lidocaine exhibited an initial burst release during the first 4 h, followed by a slower, sustained release phase. Kinetic analysis was therefore performed over the 4–60 h for lidocaine and 8–60h for BDMDAC to evaluate matrix-controlled transport. Within this region, the 1st-order model generally provided the closest agreement, with *R* ^2^ values reaching 0.9658 for GL5-Q125-L30, suggesting concentration-dependent diffusion through the hydrated gelatin network. This biphasic profile is consistent with rapid release of surface-associated drug followed by diffusion of entrapped molecules and may be advantageous clinically, as the initial burst could provide rapid onset of analgesia immediately after implantation while sustained release maintains therapeutic levels over time.

**Table 3:** Fit of Cumulative Release of BDMDAC and Lidocaine According to Various Models of Kinetic Release

		**0th order**	**1st order**	**Higuchi**
		***R*** ^**2**^		
BDMDAC	GL5-Q125	0.9372	**0.9480**	0.9091
	FGL5-Q125	**0.9346**	0.8776	0.8917
	GL5-Q125-L15	**0.9656**	0.9096	0.9388
	FGL5-Q125-L15	0.9061	**0.9087**	0.8879
	GL5-Q125-L30	0.9367	**0.9469**	0.9061
	FGL5-Q125-L30	**0.9301**	0.8685	0.8858
lidocaine	GL5-Q125			
	FGL5-Q125			
	GL5-Q125-L15	0.9534	**0.9614**	0.9020
	FGL5-Q125-L15	**0.9442**	0.9365	0.9131
	GL5-Q125-L30	0.9428	**0.9658**	0.8753
	FGL5-Q125-L30	0.8889	**0.9289**	0.8052

In contrast, BDMDAC displayed a less pronounced burst and a more gradual, prolonged release profile. 0th- and 1st-order models yielded comparable fits across gels, with similar *R* ^2^ values, preventing a definitive determination of the dominant transport mechanism. The slower release may reflect differences in molecular size and electrostatic interactions with the gelatin matrix, which could slow diffusion and promote extended retention within the gel, thereby sustaining antimicrobial activity. Higuchi fits were consistently lower across most samples, indicating that release is not governed solely by a slab-type reservoir but rather by matrix-controlled diffusion within the hydrated network.

This is consistent with prior reports describing diffusion-governed mass transport in hydrated gel systems, where solute transport is driven by concentration gradients through the polymer network.^30^ Predictable and spatially controlled transport has also been demonstrated in embedded gel constructs, with diffusion time and material microstructure regulating mass transfer.^31^ Studies of 3D-printed hydrogels with tailored internal architectures further show that release behavior can be described by 1st-order kinetics dominated by Fickian diffusion and, in some cases, matrix erosion.^32^ Modeling of comparable 3D-printed hydrogel systems has additionally demonstrated that diffusion, rather than polymer relaxation, predominates in controlling active release.^33^ Overall, our results are consistent with matrix-controlled diffusion through the hydrated network, though the kinetic fits do not allow a definitive identification of the dominant release mechanism.

### 3.3 Antimicrobial Assay (Planktonic)

The antimicrobial efficacy of both GL and FGL BDMDAC/lidocaine gels was evaluated at 4 and 24 h against three clinically relevant oral pathogens: *P. gingivalis*, *E. faecalis* , and *S. mutans* (Figure 4a–c). Across all species, the Q125 gels demonstrated the strongest antimicrobial activity. Both GL5-Q125 and FGL5-Q125 produced significant reductions in bacterial concentration at both time points, confirming the potency of BDMDAC. Comparable performance between GL and FGL indicates that antimicrobial activity is preserved irrespective of physical state and highlights the broad-spectrum efficacy of the gels.

**Figure 4:**
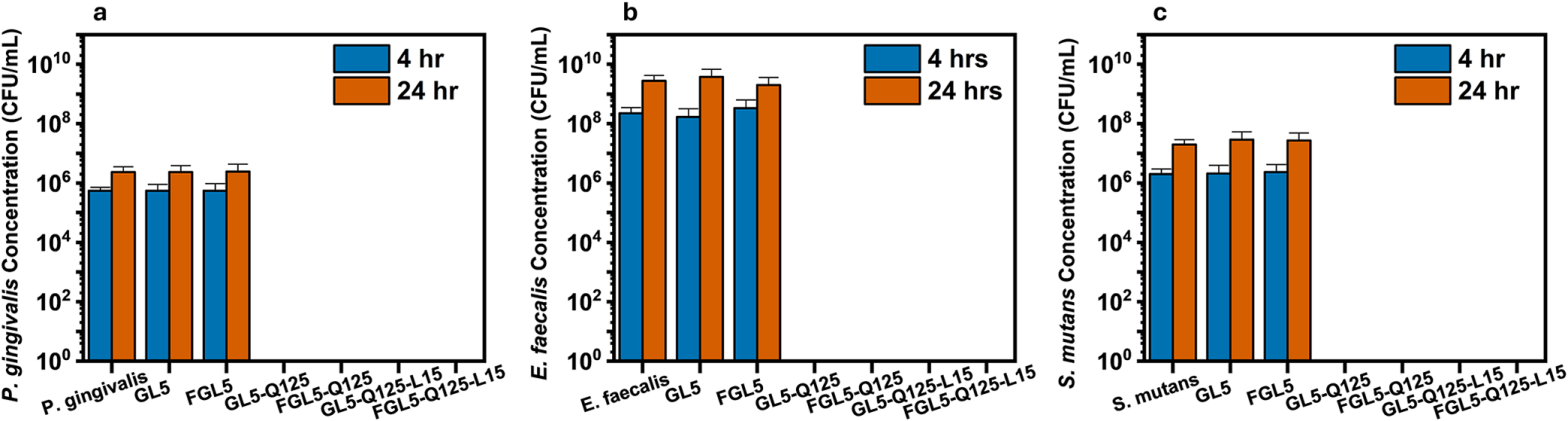
Planktonic inhibition assay of BDMDAC/lidocaine in GL and FGL gels against (a) *P. gingivalis*, (b) *E. faecalis* , and (c) *S. mutans* after 4 and 24 h. Error bars represent standard deviations, (*N* = 3, *n* = 4).

Although the precise bactericidal mechanism of quaternary ammonium compounds (QACs) remains incompletely defined, they are widely accepted to act *via* contact-mediated membrane disruption.^34^ QACs interact with the bacterial membrane, where the hydrophobic tail inserts into the lipid bilayer and the positively charged head binds to the negatively charged phosphates, ultimately causing membrane disruption, pore formation and cell death.^34^ This mechanism has been experimentally supported by Van de Lagemaat *et al*., who demonstrated similar effects against *E. faecalis* and *S. mutans*.^35^

Lidocaine was incorporated into BDMDAC formulations at lower (L15, 15 mg/mL) and higher (L30, 30 mg/mL) concentrations in GL (GL5-Q125-L15, FGL5-Q125-L15, GL5-Q125-L30), and FGL (FGL5-Q125-L30) gels. Inclusion did not result in any significant differences in bacterial reduction compared with GL5-Q125 and FGL5-Q125 alone. These results indicate that lidocaine neither enhances nor diminishes the antimicrobial activity of BDMDAC and instead functions solely in its intended role as a local anesthetic, with no measurable impact on BDMDAC’s antimicrobial mechanism. In the antimicrobial and antibiofilm assays, FGL5 Q125 and GL5 Q125 were used as the reference controls, as the antimicrobial activity of gelatin only gels (GL5 and FGL5) has been extensively characterized in our previous work.^16^ In the present study, the objective was to determine whether the incorporation of lidocaine at 15 and 30 mg/mL altered the established antimicrobial efficacy of Q125 loaded gelatin gels, rather than to validate the intrinsic behavior of gelatin based matrices.

### 3.4 Antimicrobial Biofilm Inhibition Assay

Preventing biofilm formation in the extraction socket is essential for managing AO. The biofilm inhibition assay assessed the ability of BDMDAC/lidocaine GL and FGL gels to prevent *P. gingivalis*, *E. faecalis* , and *S. mutans* biofilms over 24 h (Figure 5a–c).

**Figure 5:**
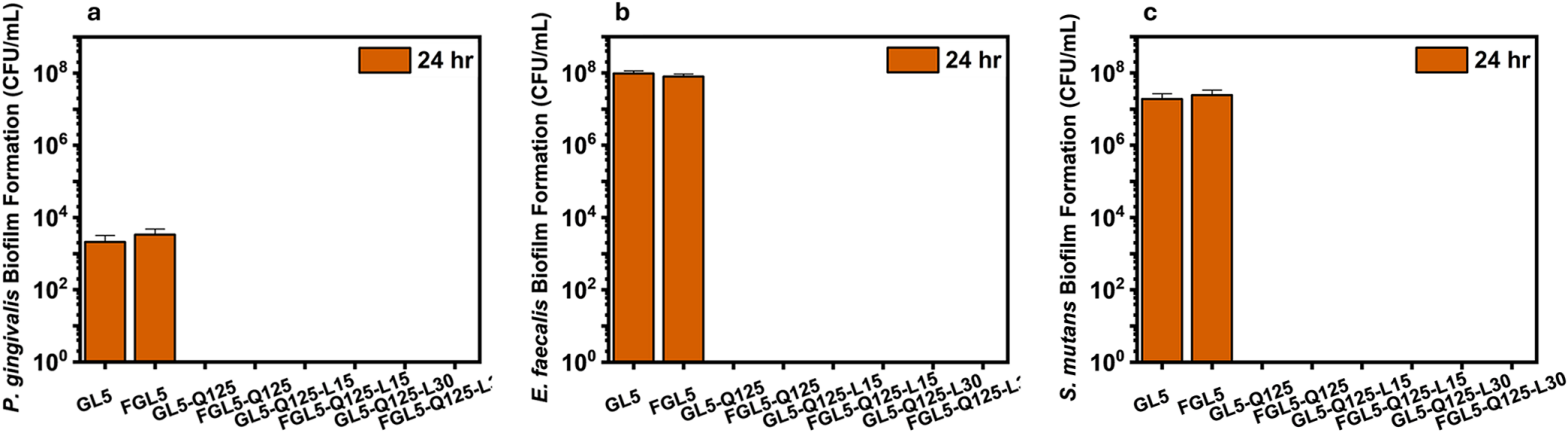
Biofilm inhibition assay of BDMDAC/lidocaine GL and FGL gels against (a) *P. gingivalis*, (b) *E. faecalis* and (c) *S. mutans* after 24 h. Error bars represent standard deviations.( *N* = 3, *n*= 4).

Across all species, BDMDAC (Q125) produced a robust and consistent antibiofilm effect. Both GL5-Q125 and FGL5-Q125 significantly reduced biofilm formation compared with their respective controls, demonstrating that BDMDAC retains its efficacy regardless of gel form. These results align with previous findings showing complete bacterial eradication at 0.125 μg/mL BDMDAC.^16^ The addition of lidocaine at 15 mg/mL (L15) or 30 mg/mL (L30) did not alter this effect; the GL5-Q125-L15, FGL5-Q125-L15, GL5-Q125-L30, and FGL5-Q125-L30 gels performed similarly to BDMDAC alone. These results indicate that lidocaine does not interfere with BDMDAC’s antimicrobial activity and instead provides local anesthesia without compromising biofilm inhibition.

Overall, BDMDAC-based gels, in both GL and FGL forms, with or without lidocaine, exhibited strong biofilm inhibitory activity against all three clinically relevant oral pathogens.

### 3.5 Cell Viability

The cytocompatiblity of GL and FGL gels with BDMDAC/lidocaine was assessed by investigating the cell viability of human gingival fibroblast cells (HGF-1) after exposure to the gels according to ISO-10993 standards. (Figure 6). The gels were all submerged in DMEM media at 37 °C for 24 h to allow BDMDAC and lidocaine to leach into the media. The GL5-Q125 and FGL5-Q125 gels at 125 μg/mL BDMDAC initially supported cell viability at 24 h (>70%). However, by 72 h, GL5-Q125 viability declined below the 70% cytotoxicity threshold defined by ISO 10993, whereas FGL5-Q125 maintained cell viability above this limit.^36^ This suggests that GL gels may exhibit reduced physical stability or altered release behavior over time, leading to transient increases in local BDMDAC exposure that could influence cell viability. Lidocaine concentration further influenced cell response. Incorporating 15 mg/mL lidocaine (GL5-Q125-L15) significantly improved viability at 72 h relative to GL5-Q125, indicating a potential protective or modulatory effect, possibly through membrane stabilization.^37^ In contrast, increasing lidocaine to 30 mg/mL (GL5-Q125-L30) reduced viability below 70%, demonstrating dose-dependent cytotoxicity. This pattern occurred in both GL and FGL gels although FGL consistently showed higher cell viability. These findings suggest that freeze-drying not only improves physical stability but may also modulate the release kinetics of BDMDAC and lidocaine in a manner that better supports cell viability.

**Figure 6:**
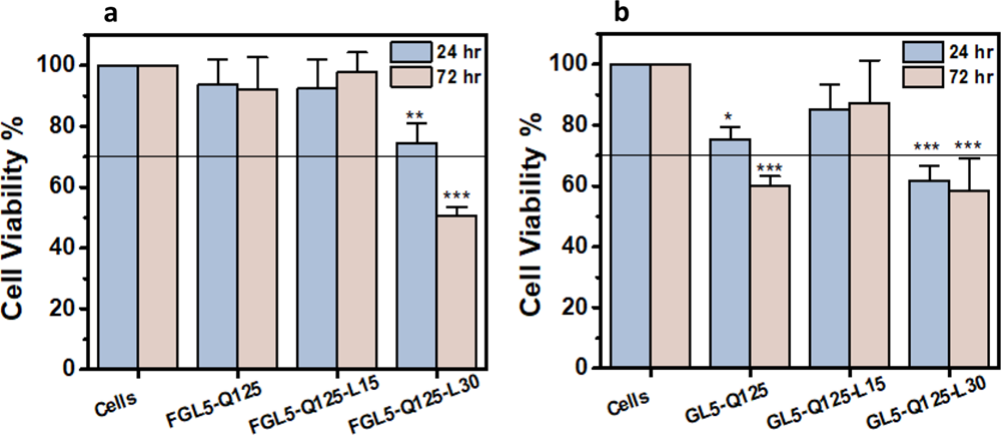
Percentage cell viability of (a) FGL and (b) GL gels containing BDMDAC (125 μg/mL) and lidocaine (L15:15 mg/mL, L30:30 mg/mL) at 24 and 72 h. The horizontal line at 70% represents the threshold for biocompatibility. * *p* < 0.1, **p < 0.001, *** *p* < 0.001, (*N* = 3, *n* = 3).

Overall, cytocompatibility depends on both lidocaine concentration and gel format. For clinical translation, an ideal lidocaine release profile should provide rapid analgesia during the first 24–48 h after application, when pain and inflammation are typically greatest. This could be achieved by delivering therapeutic levels (approximately 5–10 mg/mL)^37^ via an initial burst release, followed by a controlled sustained release to minimize cytotoxicity. Such a profile would minimize systemic exposure while maximizing localized pain control. Sustaining gel integrity and biocompatibility throughout this period is essential to prevent inflammatory responses and support tissue healing. In this study, gels such as FGL5-L30 demonstrated an initial burst release, that may provide strong early analgesia but could require further optimization to prevent transient cytotoxic effects or pro-inflammatory response.

### 3.6 Quantitation of IL-6 and TNF-α Release from HGF-1 Cells Using ELISA Assays

Inflammation plays a central role in the pathogenesis and symptom severity of AO, therefore evaluating pro-inflammatory cytokines including IL-6 and TNF-α are important for determining whether emerging therapies support healing rather than exacerbate tissue irritation. The analysis of IL-6 expression by HGF-1 cells (Figure 7a) revealed an increase across most gels. While the control group showed no significant change from 1 to 6 h, all gels with lidocaine induced a similar or increased IL-6 response. At 1 h, gels containing 30 mg/mL lidocaine (GL5-L30, FGL5-L30) produced the highest IL-6 levels. At 6 h, lidocaine no longer appeared to drive the response, as FGL5-Q125 alone showed IL-6 levels similar to lidocaine-containing groups. Indeed, BDMDAC and, gel architecture appear to also play a role. Interestingly, the FGL gels generated a significantly higher IL-6 expression than their GL counterparts, regardless of lidocaine content.^38^

**Figure 7:**
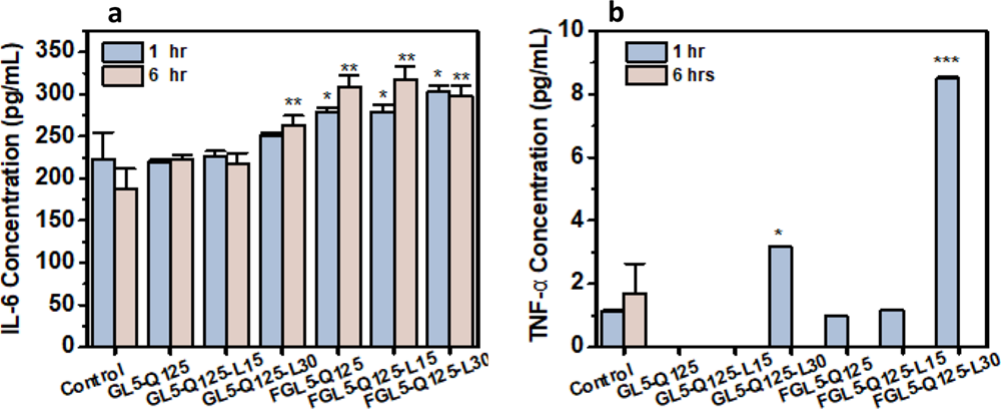
Pro-inflammatory cytokine secretion of (a) IL-6 and (b) TNF-α by HGF-1 cells in response to BDMDAC/lidocaine GL and FGL gel exposure. *N* = 3, *n* = 3, * *p* < 0.05, ** *p* < 0.01, *** *p* < 0.001.

In contrast, TNF-α expression (Figure 7b) remained low and comparable to the control across most groups. The only notable exception was the FGL5-L30 group, which produced an ∼8-fold increase in TNF-α at 1 h that returned to baseline by 6 h. This divergence between pro-inflammatory cytokines may reflect their distinct biological kinetics. TNF-α is associated with early, acute inflammatory signaling and rapidly declines due to its short half-life, whereas IL-6 plays a broader role in both acute and sustained inflammation and tends to accumulate over time.^39^ In the first hour, IL-6 expression can be beneficial as it helps recruit immune cells. Overall, this suggests that all gels, particularly FGL5-L30, may provoke an initial transient TNF-α response followed by a more prolonged IL-6 phase, with gel structure exerting a greater influence than lidocaine concentration.

### 3.7 Proof-of-Concept Patient Specific Gels

The proof-of-concept for patient-specific gels was demonstrated by fabricating anatomically relevant, molar-shaped gels for both adult and pediatric shapes. Drug loading was optimized to maintain antimicrobial and analgesic efficacy across different sizes, and these gels were evaluated for their ability to deliver consistent therapeutic outcomes while preserving their physicochemical properties. As shown in Figure 8a–c, the fabrication of molar-shaped gels highlights the versatility of the printing process and its potential for personalized clinical use. Figure 8 presents the complete design-to-print workflow, CAD modeling (Figure 8a), slicing (Figure 8b), and final printing of the adult molar gel (Figure 8c).

**Figure 8:**
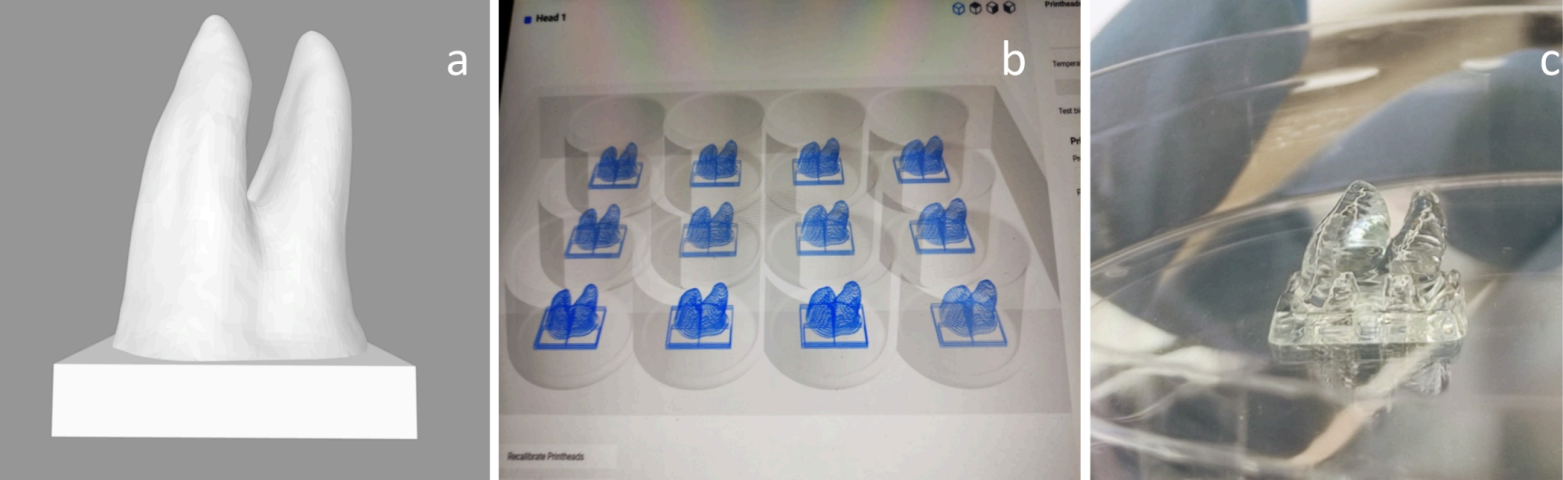
(a) CAD design, (b) sliced view in the printing software (c) and printed adult molar gel

Transitioning from a simple grid design to the molar geometry did not affect antimicrobial performance. Complete eradication of *P. gingivalis*,*E. faecalis*, and *S. mutans* was observed at both 4 h (Figure 9a–c) and 24 h (Figure 9d–f), along with full biofilm inhibition at 24 h (Figure 10). These outcomes indicate that antimicrobial efficacy is independent of shape, provided that key parameters, including total drug mass, filament spacing, infill density, and cross-linking conditions are maintained. The comparable results also suggest that diffusion and release of the antimicrobial agent were not hindered by the more complex architecture. Consistent with the behavior observed in Section 3.2 drug transport could be governed by hydration of the gelatin network and concentration gradients rather than macroscopic scaffold geometry. Provided that porosity and filament dimensions are preserved, similar swelling profiles and matrix permeability are expected, enabling comparable release kinetics despite changes in shape. This will be investigated in a larger follow-up study. Experimental group nomenclature is summarized in Table 4.

**Table 4:** Nomenclature of the Adult and Pediatric Molar-shaped Gels

**sample name**	**BDMDAC**concentration (μg/mL)	**lidocaine** **concentration**(mg/mL)
adult molar (BGL5)		
BQ125	125	
BL15	125	15
BL30	125	30
pediatric molar (SGL5)		
SQ125	125	
SL15	125	15
SL30	125	30

**Figure 9:**
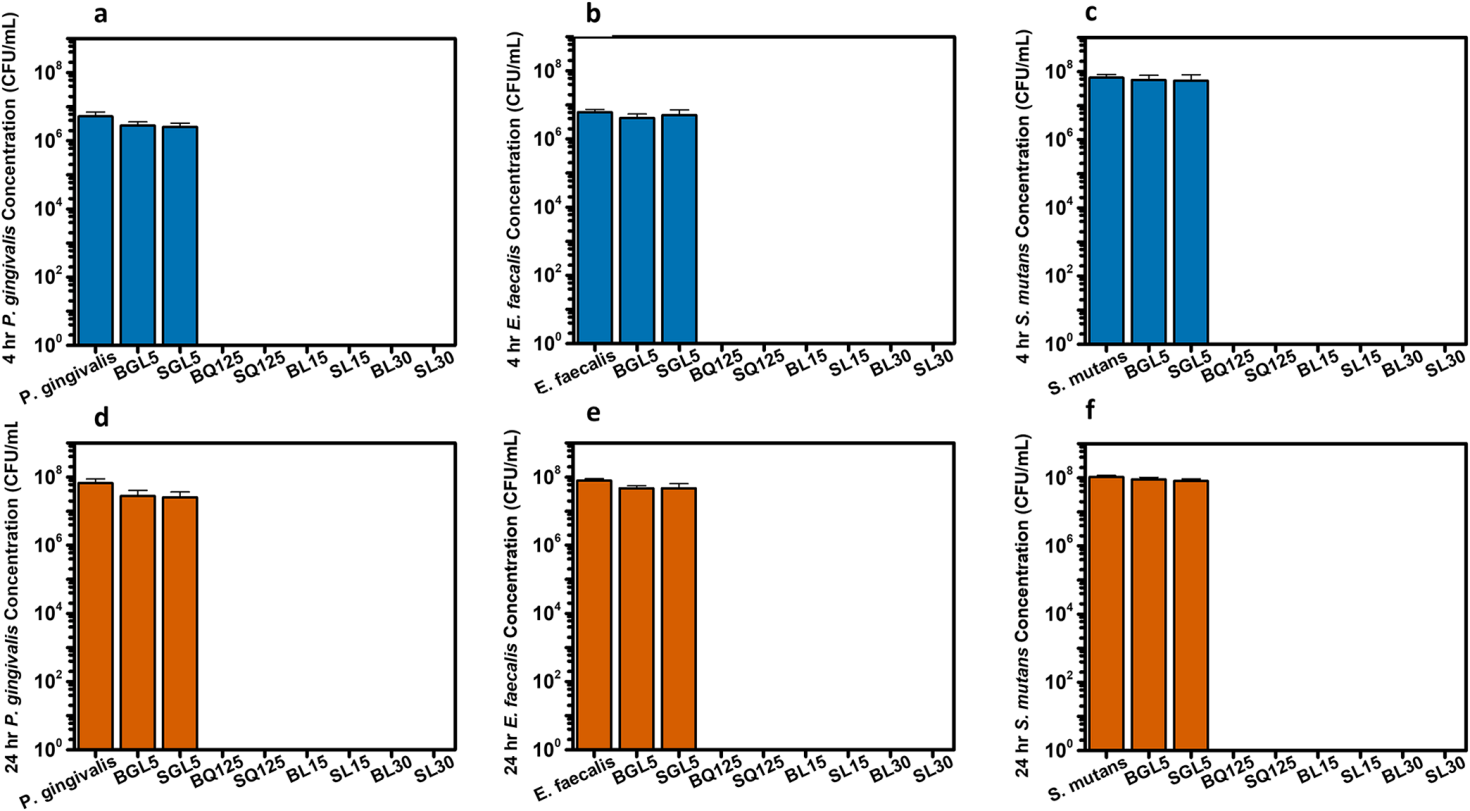
Planktonic inhibition assay of BDMDAC/lidocaine anatomic molar-shaped gels against*P. gingivalis* at (a) 4 h and (d) 24 h, *E. faecalis* at (b) 4 and (e) 24 h and*S. mutans* at (c) 4 h and (f) 24 h. (*N = 3,* *n* = 4)

**Figure 10:**
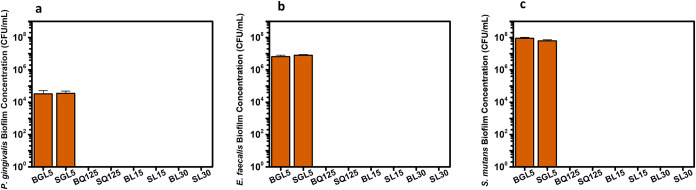
Biofilm inhibition assay of BDMDAC/lidocaine anatomical molar shaped gels against (a) *P. gingivalis*, (b) *E. faecalis* (c), and *S. mutans* after 24 h. (*N = 3,* *n* = 4)

Although bulk gel geometry can influence drug release behavior by altering the exposed surface area-to-volume ratio and diffusion path lengths, in this study, quantitative release experiments were intentionally performed using standardized grid architectures to isolate the effect of gel composition on drug transport and antimicrobial performance. Maintaining a consistent and simplified geometry minimized variability arising from macroscopic structural differences and enabled direct comparison between gels under controlled conditions. The anatomically relevant adult and pediatric molar geometries presented here serve primarily as a proof-of-concept demonstration of patient-specific fabrication and anatomical conformity rather than as quantitative drug release models. A systematic evaluation of geometry-dependent release kinetics, including normalization for surface area and volume, represents an important direction for future investigation.

### 4 Conclusion

This work establishes 3D-printed gelatin-based gels incorporating BDMDAC and lidocaine as a robust, dual-function material platform for the potential management of alveolar osteitis. The integration of antimicrobial and analgesic agents within a structurally stable, printable gel matrix, enables localized infection control and sustained pain relief from a single, customizable gel. BDMDAC provided potent and sustained antimicrobial activity against clinically relevant oral pathogens *P. gingivalis*, *E. faecalis* and *S. mutans*, achieving complete biofilm inhibition and pathogen eradication within 24 h, while lidocaine incorporation did not compromise antimicrobial performance and provided analgesia.

The gels demonstrated good cytocompatibility with HGF-1 cells and showed a minimal inflammatory response, evaluated by IL-6 and TNF-α expression, supporting their suitability for clinical translational. Importantly, anatomical patient-specific molar-shaped gels preserved antimicrobial efficacy when compared to grid-printed controls, confirming that therapeutic performance is maintained across complex geometries. Control over printing and formulation parameters, including mass, filament spacing, infill density, and cross-linking enabled tuning of therapeutic drug loading, release profiles, and bioactivity, Overall, these results demonstrate that on-demand fabrication of patient-specific, antimicrobial and analgesic 3D-printed gels has the potential overcome key limitations of conventional AO treatments.

## Supplementary Material



## Notes

Supporting information includes rheological analysis, filament collapse test, diffusion rates, and printability (PDF)
