# Supplementary material for: Patient-Specific Dual-Function 3D-Printed Gels for Antimicrobial and Analgesic Management of Alveolar Osteitis
Source: ACS Mater Au. 2026 Apr 6;6(4):705–15. doi: 10.1021/acsmaterialsau.5c00226 (PMC13352265; doi:10.1021/acsmaterialsau.5c00226)
Supplement: Supplementary file 1 [file mg5c00226_si_001.pdf]

Patient-Specific Dual-Function 3D-Printed Gels for Antimicrobial and Analgesic Management of Alveolar Osteitis

Mateo Dallos Ortega[1], Vahid Heravi Shargh[1], Jenny Aveyard[1], Mark Hunter[1], Alexander Ciupa[2], and Raechelle A. D'Sa[1*]

[1]School of Engineering, University of Liverpool, Harrison Hughes Building, Brownlow Hill, Liverpool, L69 3GH, UK

[2]Materials Innovation Factory, University of Liverpool, 51 Oxford Street, Liverpool, L7 3NY, UK

S1. Rheological Analysis

The rheological properties of the inks were assessed using an HTR 502 modular compact rheometer (Anton Paar, Ostfildern, Germany) fitted with a 40 mm diameter parallel rough plate and a solvent trap to minimize hydrogel evaporation. The rough plate was lowered to a 1 mm gap height, and any excess ink was removed. Measurements were performed in triplicate, with the results reported as the average of three tests. A temperature sweep was carried out at 0.5% strain amplitude and a frequency of 1 Hz, gradually cooling the samples from 40 °C to 15 °C at a rate of 1 °C/min. Strain amplitude sweep tests were then conducted to determine the linear viscoelastic region, using a strain range of 0.1–1000% at a constant frequency of 1 Hz. This was followed by frequency sweep tests at 0.5% strain, spanning a frequency range of 0.1 to 10 Hz. The complex viscosity (η*) was calculated based on the storage modulus (G') and loss modulus (G'') obtained from the frequency sweep test. All measurements were taken at room temperature (21 °C).

A comprehensive understanding of the gelatin and gelatin-QAC/lidocaine biomaterials inks in terms of their rheological characteristic was conducted prior to printing using oscillatory shear. The rheology was measured in terms of amplitude sweep (Figure S1 (a)), frequency sweep (Figure S1 (b)), temperature dependence (Figure S1 (c)), and complex viscosity as a function

of frequency (Figure S1 (d)). The results from these tests provide insights into the inks' flow properties, structural stability, and response to temperature and frequency variations.

GL5 serves as the base ink and exhibits the lowest viscosity and moduli (G', G") among the four formulations, indicating that it is the least resistant to deformation. The addition of Q125, L15, and L30 modifiers progressively increases both the viscosity and moduli, suggesting enhanced internal structure and increased resistance to flow. The amplitude and frequency sweep tests show the storage modulus (G') exceeds the loss modulus (G"), indicating that all inks exhibit more solid-like than liquid-like behaviour under the tested conditions.[1, 2] In the amplitude sweep test, all inks show a plateau region at low strain amplitudes where G' and G" remain relatively constant, known as the linear viscoelastic region (LVR), where the material's microstructure is not significantly disrupted by the applied strain. This LVR extends to higher strain amplitudes for the modified inks compared to GL5, demonstrating that the modifications enhance the material's structural stability. The crossover point, where G' and G" intersect, marks the transition from solid-like to liquid-like behaviour, and this point shifts to higher strain amplitudes with the addition of modifiers, further emphasizing their strengthening effect. In the frequency sweep, both G' and G" increase with frequency for all inks, confirming viscoelastic behaviour typical of polymeric materials, where the response depends on the timescale of deformation.[1, 2] At low frequencies, G' reaches a plateau, representing the material’s long-time relaxation modulus, and the magnitude of this plateau increases with the addition of modifiers, suggesting the formation of a more robust network structure. In the temperature dependence tests, all inks show a decrease in G' and G" with increasing temperature, indicative of thermal softening, as higher temperatures provide more thermal energy to the molecules, facilitating movement and reducing the material's resistance to deformation.[1, 2] Notably, the curve GL5-Q125-L30 shows a more pronounced transitions in the tested temperature range compared to GL5, GL5-Q125, and L15 suggesting that the

concentration of 30 mg/ml of lidocaine would be more sensitive at undergoing phase transitions or structural changes with temperature. In the complex viscosity tests, all inks exhibit shear thinning behaviour, where the complex viscosity decreases with increasing frequency, consistent with the amplitude sweep results and further confirming the non-Newtonian nature of the inks. The zero-shear viscosity, representing the viscosity at very low frequencies, increases with the addition of modifiers, indicating a heightened resistance to flow. Specifically, the addition of Q125 to GL5 (GL5-Q125) increases the viscosity and moduli compared to GL5, suggesting that Q125 enhances intermolecular interactions or forms a more entangled network.[3] However, the addition of lidocaine does not significantly alter the viscosity of Q125, further supporting the role of Q125 in modifying the material's structural properties without affecting its flow behaviour.

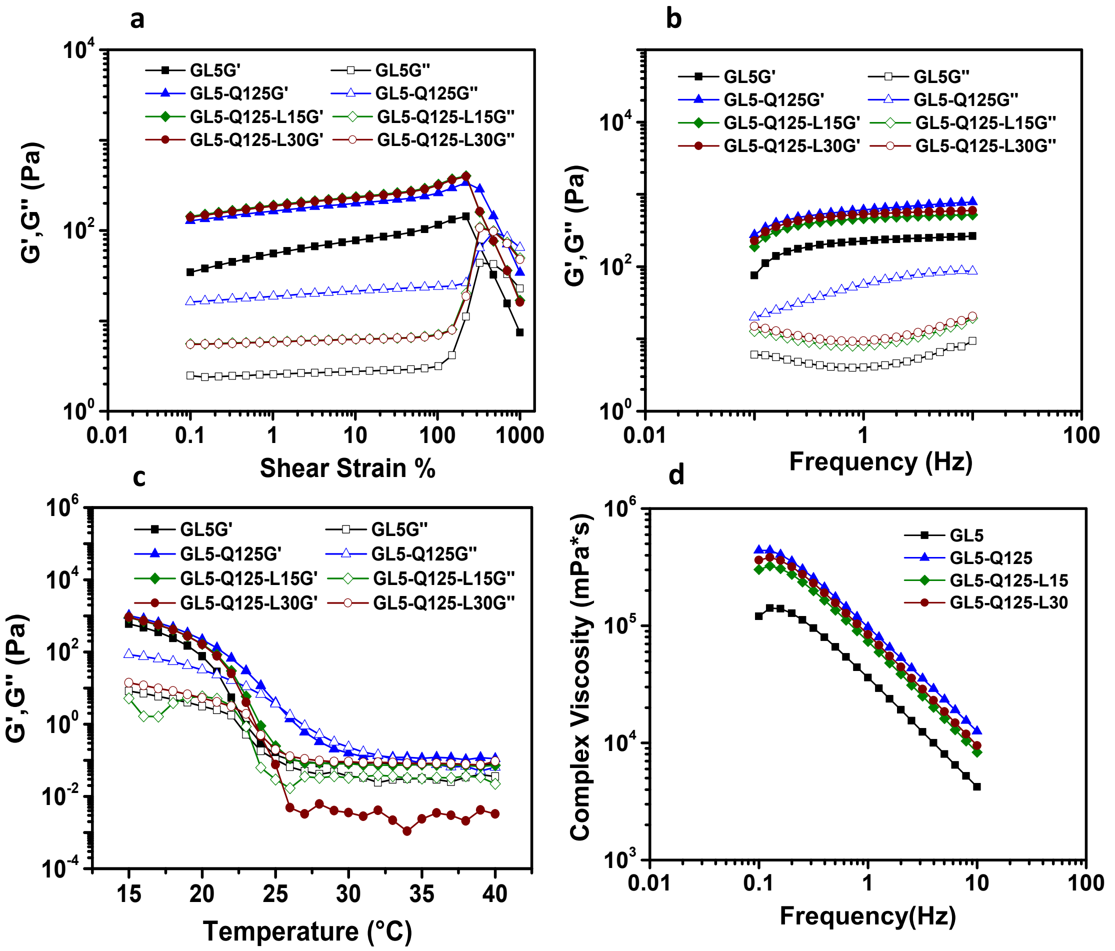

Figure S1: Comprehensive rheological characterization of gelatin/BDMDAC/lidocaine inks. (a) Amplitude sweep test, evaluating the storage (G') and loss (G'') moduli as a function of strain to determine the linear viscoelastic region (LVR). (b) Frequency sweep test, illustrating the dependence of G' and G'' on oscillatory frequency to assess the structural stability of the ink. (c) Temperature dependence sweep, showing variations in viscoelastic properties with temperature changes, crucial for thermal stability analysis. (d) Viscosity as a function of frequency, highlighting the shear-thinning behaviour of the ink. Data represent the mean ± standard deviation for N = 3 independent measurements.

S2. Filament Collapse Test, Diffusion Rate and Printability

The filament collapse test was performed following our previous work[4] and the studies by Habit et al.[5] and Therriault et al.[6] This test evaluates filament stability by measuring mid-span deflection after extrusion onto a designated stage.

The stage was designed in Creo Parametric® and featured seven pillars with increasing gap distances, ranging from 1 mm to 6 mm in 1 mm increments. The middle pillars measured 2 ×

10 × 6 mm (W x H x L), while the corner pillars were 5 × 10 × 6 mm (W x H x L). The design was printed using an Up Mini 2 3D printer with polylactic acid (PLA) filament.

A single filament of ink was deposited onto the platform, and a Sony IMX 682 64 MP 1/1.73″ camera was used to immediately capture an image, minimizing the impact of time-dependent deflection. The pressure, temperature, extrusion velocity, and nozzle diameter were kept consistent with the scaffold printing parameters.

The collapse area factor (C_f) was determined using the following equation:[5, 7, 8]

$$C_f(\%) = 100 - (\frac{Real\ Area\ (A_a)}{Theoretical\ Area\ (A_t)} \times 100) \qquad (1)$$

In Equation (1), the theoretical area represents the ideal rectangular area defined by straight lines connecting the support points, assuming the filament retains its intended shape without sagging. In contrast, the real area is the projected area measured from the printed filament, using the deflected centreline as the base of the rectangle. This approach captures the degree of collapse caused by gravitational deformation during deposition. If the ink is too dense to bridge between two pillars, the actual area is considered zero, resulting in a collapse area factor of 100%. Conversely, if the ink successfully forms a bridge, the actual and theoretical areas will be identical, yielding a collapse area factor of 0%.

For the diffusion rate test, scaffolds consisting of two consecutive layers were printed in a 0–90° pattern, with the spacing between adjacent filaments increasing incrementally from 1 mm to 5 mm. To minimize the effects of time-dependent deformation, images of the printed scaffolds were captured immediately after fabrication using a Sony IMX 682 64 MP 1/1.73″ camera. Printing speed, temperature, pressure, and nozzle diameter remained consistent with the previously established parameters.

The diffusion rate (Df_r) and printability (Pr) were calculated using the following equations:[5, 7, 8]

$$Df_r = \frac{PA_t - PA_r}{PA_t} \times 100 \quad (2)$$

$$\mathrm{Pr} = \frac{L^2}{16PA_r} \quad (3)$$

where **PA**□ is the *theoretical pore area* (i.e., the ideal square area based on filament spacing), and **PA**$_r$ is the *real pore area* measured from the printed scaffold. **L** is the measured perimeter of the actual printed pore. A diffusion rate of 0% indicates no spreading (perfect fidelity), while a printability value of 1 corresponds to an ideal square-shaped pore.

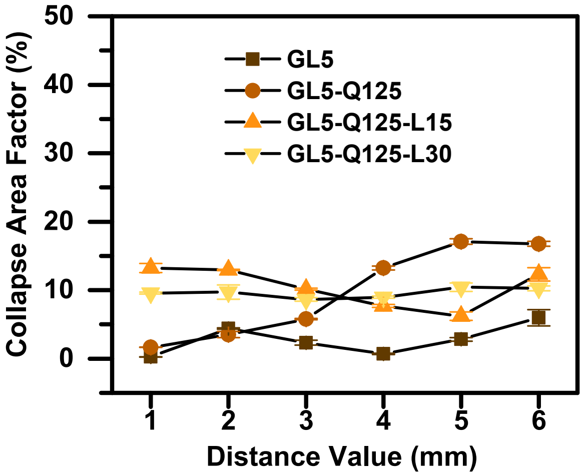

Figure S2: Collapse area factor (%) as a function of distance (mm) for four ink formulations (GL5, GL5-Q125, GL5-Q125-L15, and GL5-Q125-L30).

Figure S2 represents the collapse area factor (%) as a function of distance (mm) for four different ink formulations: pure gelatin (GL5), gelatin with BDMDAC (GL5-Q125), and gelatin with BDMDAC combined with two different concentrations of lidocaine (15 mg/mL: GL5-Q125-L15 and 30 mg/mL: GL5-Q125-L30). The collapse area factor quantifies the extent of structural deformation in the material, with higher values in distance indicating greater

susceptibility to collapse. Pure gelatin (GL5) exhibits the lowest collapse area factor across all distances, suggesting that it maintains a more stable structure under the given conditions, likely due to its natural gelation properties and inherent network stability.[9] For GL5-Q125, the incorporation of BDMDAC increases the collapse area factor across all distances, indicating that BDMDAC alters the structural integrity of gelatin. This effect may be attributed to BDMDAC's surfactant nature[10], which could modify the network interactions within the gelatin matrix, making it more susceptible to deformation. In the inks containing lidocaine (GL5-Q125-L15 and GL5-Q125-L30), the collapse area factor is generally higher than that of pure gelatin (GL5) but similar to GL5-Q125. Notably, increasing the lidocaine concentration from 15 mg/mL to 30 mg/mL does not significantly increase the collapse area factor. This suggests that lidocaine incorporation does not further destabilise gelatin's structure[11] beyond the effect already caused by BDMDAC.

Figure S3 (a) illustrate the diffusion rate (%) and printability ratio (b) for four ink formulations (GL5, GL5-Q125, GL5-Q125-L15, and GL5-Q125-L30) across different grid spacings. The diffusion rate is highest at 1×1 mm^2, where all formulations exhibit nearly 100% spreading, indicating a lack of structural retention. As grid spacing increases, diffusion significantly decreases, stabilising at lower values beyond 3 × 3 mm^2, suggesting improved structural integrity. There is no statistically significant difference in diffusion rates between formulations, meaning the addition of BDMDAC or lidocaine does not substantially alter ink spreading behaviour. Notably, at 1 × 1 mm^2, the printability ratio is effectively zero, indicating that none of the formulations can maintain a defined structure at this grid spacing due to excessive spreading. However, as grid spacing increases, the printability ratio stabilizes close to 1.00 for all formulations, demonstrating excellent structural fidelity regardless of composition. This suggests that while diffusion behaviour varies with grid spacing, printability remains stable beyond 2 × 2 mm^2, making these suitable biomaterial inks.

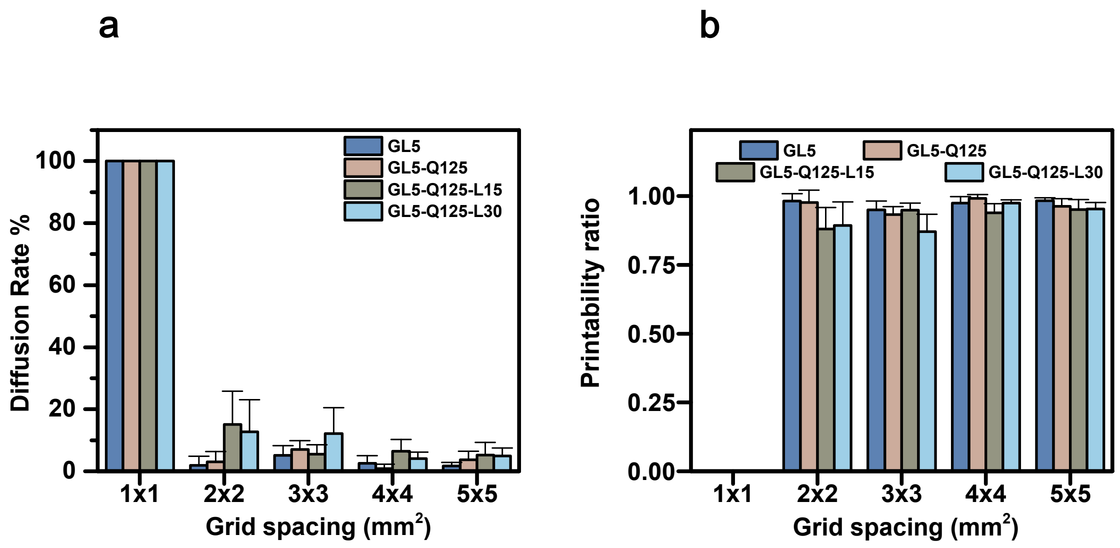

Figure S3: Diffusion rate (%) (right) and printability ratio (left) of four ink formulations (GL5, GL5-Q125, GL5-Q125-L15, and GL5-Q125-L30) across different grid spacings.

References

1. Rau, D.A., M.J. Bortner, and C.B. Williams, *A rheology roadmap for evaluating the printability of material extrusion inks.* Additive Manufacturing, 2023. **75**: p. 103745.
2. Bercea, M., *Rheology as a Tool for Fine-Tuning the Properties of Printable Bioinspired Gels.* Molecules, 2023. **28**(6).
3. Meng, X., et al., *Viscosity of Gelatin Solution Containing Cationic Crosslinker.* Advanced Materials Research, 2013. **750-752**: p. 1660-1663.
4. Mateo Dallos Ortega, J.A., Alexander Ciupa, Robert J Poole, David Whetnall, and Raechelle A. D'Sa, *Printable gelatin/nisin antimicrobial hydrogel ink for 3D bioprinting and tissue engineering applications.* 2024.
5. Habib, A. and B. Khoda, *Development of clay based novel hybrid bio-ink for 3D bio-printing process.* Journal of Manufacturing Processes, 2019. **38**: p. 76-87.
6. Therriault, D., S.R. White, and J.A. Lewis, *Rheological Behavior of Fugitive Organic Inks for Direct-Write Assembly.* Applied Rheology, 2007. **17**(1): p. 10112-1-10112-8.
7. Ribeiro, A., et al., *Assessing bioink shape fidelity to aid material development in 3D bioprinting.* Biofabrication, 2017. **10**(1): p. 014102.
8. Gillispie, G., et al., *Assessment methodologies for extrusion-based bioink printability.* Biofabrication, 2020. **12**(2): p. 022003.
9. Alipal, J., et al., *A review of gelatin: Properties, sources, process, applications, and commercialisation.* Materials Today: Proceedings, 2021. **42**: p. 240-250.
10. Hinchliffe, D., et al., *The adsorption of alkyl-dimethyl-benzyl-ammonium chloride onto cotton nonwoven hydroentangled substrates at the solid-liquid interface is minimized by additive chemistries.* Textile Research Journal, 2015. **87**.

11. Bahmani, S., et al., *Transdermal drug delivery system of lidocaine hydrochloride based on dissolving gelatin/sodium carboxymethylcellulose microneedles.* AAPS Open, 2023. **9**(1): p. 7.
